# Meaning Matters: Self-Perceived Meaning in Life, Its Predictors and Psychological Stressors Associated with the COVID-19 Pandemic

**DOI:** 10.3390/bs11040050

**Published:** 2021-04-13

**Authors:** Ashley Humphrey, Olivia Vari

**Affiliations:** School of Science, Psychology and Sport, Federation University, Mount Helen 3350, Victoria, Australia; olivia.vari@gmail.com

**Keywords:** COVID-19, coping, meaning in life, psychological wellbeing, intrinsic aspirations

## Abstract

Past research has found that a perceived meaning in life can act as a protective factor against adverse mental health symptomology, while also providing coping resources to buffer against the impact of negative life events. The current research investigated how the impact of self-perceived meaning in life as well as its predictors interact with stressors and worry related to the COVID-19 pandemic. We collected survey based data (*n* = 260) from Australian participants during the pandemic, measuring their meaning in life, orientation to differing life goals and COVID-19 related stressors via the impact of events scale. We found that meaning in life predicted less stress and worry associated with COVID-19. We also found that intrinsic based aspirations related positively to meaning in life within this context whereas extrinsic based goals related negatively to it, although these aspirations were not significant in reducing the stressors associated with COVID-19. These results reinforce past findings that meaning in life can effectively buffer against the impact of negative life events such as the COVID-19 pandemic. They also suggest that intrinsic based aspirations centred on relationships and self-acceptance may be an important mechanism in how people choose to pursue life meaning during uncertain life events.

## 1. Introduction

Along with posing a very real threat to people’s health and wellbeing, the coronavirus disease-19 (COVID-19) pandemic has generated a context of extreme uncertainty and resultant stress worldwide. Contributing to this environment of worry includes a range of extraordinary measures being implemented across the globe in order to contain the spread of the virus such as social distancing and stay at home guidelines. These measures have led to a challenging social and economic climate by way of reducing people’s opportunity to interact socially while also disrupting people’s livelihoods. From a psychological health perspective, these impacts have been noted as invoking increased feelings of fear, stress and anxiety [1,2]. Emerging attention has therefore been given to how people have buffered such stressful events via differing coping and resilience mechanisms [3,4,5].

One such avenue that has featured in past research focusing on coping with challenging circumstances is people’s sense of meaning in life, a personal framework acknowledged as relating positively to one’s psychological wellbeing [6,7,8]. Meaning in life can be conceptualised in terms of an individual pursuing and attaining personally important goals, which offer a sense of fulfilment and purpose [9,10]. An increasing body of research shows people who report a high sense of meaning in life are less likely to report symptoms of depression and anxiety [11] and more likely to report improved satisfaction with life and happiness outcomes [12]. Past findings have also associated meaning in life with greater levels of hope and optimism [13] and higher levels of resilience [14] as well as competence, determination and social integration [15].

Interestingly, residents of poorer nations have been shown to report more meaning in life than those of wealthy nations. For instance, cross-cultural findings from Oishi and Diener [16] showed that those living in low GDP (Gross Domestic Product) countries such as Ethiopia, Laos and Senegal reported far higher scores on items measuring meaning in life than responses from those living in wealthier countries such as Denmark, Belgium and Australia. Along with the increased influence of religion in developing nations versus wealthier nations, this may be in part due to the supposition that people can construct life meaning from negative events and difficult life circumstances [17,18]. As our study was conducted within Australia, such findings are suggestive that meaning in life may be of lower importance for our Australian participants comparative to those living in other contexts. This poses an intriguing aspect to the present research; with the COVID-19 pandemic bringing with it an unprecedented economic downturn [19], the need for life meaning may have been exacerbated in high GDP contexts such as Australia. Indeed, as well as helping to construct meaning from difficult situations, a strong sense of meaning in life itself is shown to be especially poignant in safeguarding one’s psychological wellbeing during times of crisis and uncertainty [3,4,20,21]. Within difficult and stressful circumstances, meaning in life has been shown to be a crucial mechanism of resilience and coping aptitude, enabling individuals to draw strengths and insights from their experiences, gain perspectives in current situations and provide a pathway towards a worthwhile and valuable future [22].

Further cultural research suggests how people choose to pursue meaning in life may have shown a shift within Western contexts over recent generations [23]. Reviewing longitudinal data from American college students collected over a 30 year period, Twenge and Kasser [24] showed a significant ‘generational shift’ in American people’s changing life goals across this time frame. Their results showed that American young people today are significantly more oriented towards extrinsic based aspirations (i.e., those that depend on the recognition of others) such as the desire for expensive possessions, money and acquiring a high paying job than the youths of the 1970s. They point to cultural influences specific to American society (and Western society more generally) as being responsible for this, citing increasing economies, high advertising penetration and the emphasis on celebrity culture as important contributors.

In light of this, we wanted to include an exploratory element to our analysis investigating how differing categories of aspirations may make up meaning in life, and whether these life aspirations may have differing outcomes in mediating the effect meaning in life has on the level of stress and worry people may experience within the context of a global pandemic. Specifically, we wanted to explore the role intrinsically oriented aspirations—defined as aspirations centred on self-acceptance, community involvement and emotional intimacy—may have in successfully mediating the role meaning in life has on wellbeing amidst the COVID-19 pandemic. These are in contrast to extrinsic based goals that are focused on gaining other people’s recognition such as acquiring status, financial success and physical attractiveness [25]. Research has shown people with a high orientation towards intrinsic based goals tend to experience better psychological wellbeing whereas those oriented towards extrinsically oriented goals are shown to experience greater psychological maladjustment [25,26]. These differing outcomes specific to wellbeing are theorised to be the results of intrinsically based goals allowing for more experiences that satisfy one’s psychological needs as opposed to extrinsic based goals that do not. We believe it to be particularly interesting to explore these aspirations and how they relate to meaning in life in such a context whereby the pursuit of both types of goals are challenged due to the restrictions in place to contain the spread of the virus.

## 2. The Current Study

The current study aimed to examine how meaning in life along with its possible predictors including purpose and self-efficacy as well as intrinsic and extrinsic values may safeguard against adverse emotional and cognitive reactions to the COVID-19 pandemic. We hypothesised that high levels of meaning in life, purpose and self-efficacy would relate positively to reducing the stressors and negative impact associated with COVID-19. Due to its focus on innate psychological needs, we also predicted that intrinsic based aspirations would associate positively with meaning in life and, as a result, partially mediate the relationship between a perceived meaning in life and the impact of COVID-19 while extrinsic aspirations—with their focus on other people’s opinions such as popularity, financial success and physical attractiveness—would not.

## 3. Methods

A total of 330 Australian participants were recruited to complete an online survey. Recruitment took place online via Australian survey distribution websites during the COVID-19 pandemic between June and September of 2020. The initial Qualtrics survey page was the plain language statement, which indicated informed consent was implied. Participants were required to be between 18 and 65 years of age, Australian citizens and English speaking. As a general sample was sought, there was no other exclusion criteria for participation. Of the 330 participants, 72 respondents were deleted as they (a) took less than three minutes to complete the survey (*n* = 43) or (b) failed to answer at least half of the survey items (*n* = 29) leaving 260 (223 females and 36 males; one did not specify) completing the survey. All studies were approved by a University ethics committee and were conducted in accordance with APA ethical conduct of research with human subjects. The sample size was considered significant to determine novel correlational relationships and also greatly exceeded the required size for running simple regression analyses with four predictor variables in each model [27]. Participants ranged from ages 18 to 65 (*M_age_* 32.20, SD 5.40). Of those participants, 36.7% reported having an average income, 28.5% above average, 5.9% well above average, 23.2% below average and 5.5% well below average. A total of 27.6% responded that they were religious while 72.4% did not associate with having a religion. Participants were asked to respond to an online questionnaire including the measures outlined below. The survey took about 15 min to complete.

**Meaning in life.** Positive life regard and the degree of meaningfulness experienced in one’s life was measured by the Life Regard Index (LRI), Adjusted Version [28]. The Life Regard Index is a 28-item questionnaire that is composed of two subscales: Framework (cognitive) and Fulfilment (affective) [9]. As advocated for in previous research, we chose to only use the Framework subscale of the LRI in our analysis, believing that the Fulfilment subscale relates to items of life satisfaction, happiness and positive affect and therefore was not central to our research interests of self-perceived meaning in life [29]. The framework subscale used has 14 items including: “I feel like I have found a really significant meaning for leading my life” and “I get completely confused when I try to understand my life” (reversed score item). Participants were asked to indicate which alternative best described their own opinion of the items on a three-point Likert Scale (I agree; I have no opinion; I disagree; *M* = 2.01 *SD* = 0.18, α = 0.96). The scores were summed with higher scores indicating a higher life regard.

**Purpose.** The sense of purpose of participants was measured via the Purpose In Life Scale, a subscale of the Psychological Wellbeing Scale [30]. The scale has five items such as “I enjoy making plans for the future bringing them into reality” and “I have a good sense of what it is I’m trying to accomplish in life”. The scale is rated on a seven-point Likert scale (1 = Strongly Agree; 7= Strongly Disagree; *M* = 4.35, *SD* = 1.10, α = 0.85). The scores for the items were summed with higher scores indicating a stronger sense of purpose in life.

**Subjective distress due to the impact of COVID-19.** Subjective distress resulting from exposure to major life events was measured by the Impact of Event Scale [31]. For the purposes of this study, the original scale was used instead of the Revised Impact of Event Scale [32] as the study only wanted to assess the original intrusive and avoidance subscales and not hyperarousal symptomology as we did not deem these items relevant to the COVID-19 pandemic. The scale has 15 items. Participants were asked to read each item and then indicate how distressing each difficulty had been for them during the past seven days with respect to COVID-19. They were then asked to rate statements such as “I had trouble staying asleep” and “I had waves of strong feelings about it” on a five-point Likert scale (0 = Not at all; 4 = Extremely; *M* = 2.24, *SD* = *0*.83, α = 0.92). The scores for the items were summed with higher scores indicating a stronger COVID-19 related stress score.

**Self-efficacy.** Self-efficacy was measured by the New General Self-Efficacy Scale [33], which assesses how much people believe they can achieve their goals despite difficulties. The measure has eight items such as “I believe I can succeed at almost any endeavour to which I set my mind”. Participants were asked to rate each statement on a five-point Likert scale (1 = Strongly Disagree; 5 = Strongly Agree; *M* = 3.80, *SD* = 0.72, α = 0.92). The responses were summed with higher scores indicating higher self-efficacy.

**Intrinsic and Extrinsic Aspirations.** To measure the aspirations of participants for the future, the Aspiration Index was used [34]. The Aspiration Index (AI) comprises 35 items measuring people’s life goals via either intrinsic or extrinsic aspirations via the subscales of Relationships, Community, Health and Personal Growth (in intrinsic aspirations) as well as Wealth, Fame and Image (extrinsic aspirations). In the original scale, participants rate (1) the importance to themselves of each aspiration, (2) their beliefs about the likelihood of attaining each and (3) the degree to which they have already attained each. In the current study, only (1) the importance to themselves of each aspiration was used. Participants were therefore asked to respond to the statements on a six-point Likert scale (1 = Not at all, 6 = Very) with items including “How important is this goal to you: To have good friends that I can count on” (intrinsic item), “To be a wealthy person” (extrinsic item); “To work for the betterment of society” (intrinsic item) and “To have my name known by many people” (extrinsic item). The scale was scored by calculating the subscale scores for each of the aspiration categories and averaging the responses of the items in that subscale (extrinsic aspirations: *M* = 2.35, *SD* = 0.85, α = 0.92; intrinsic aspirations *M* = 5.05, *SD* = 0.64, α = 0.89).

## 4. Results

Data was firstly uploaded to SPSS Version 26 for analysis and a series of correlation and regression analyses were then run. The tests of general assumptions of linear regression showed all measures were normally distributed according to the tests of kurtosis and skewness and within acceptable bounds for parametric testing at *p* < 0.05. Zero-order correlations were firstly used to examine the initial relationships between the variables. These revealed that meaning in life was overall negatively associated with stressors related to the impact of COVID-19 while also positively associated with measures hypothesised to relate positively to it including purpose and self-efficacy (see Table 1). Intrinsic aspirations were associated with an increased meaning in life as well as purpose and self-efficacy but were unrelated to the impact of COVID-19. Extrinsic based aspirations, on the other hand, were not significantly associated with either meaning in life or the impact of COVID-19.

As part of our aim was to explore potential predictors of a meaningful life, a multiple regression analysis was run in order to explore whether the independent variables of purpose and self-efficacy as well as intrinsic and extrinsic aspirations may be predictors of a meaningful life (a summary of these results is shown in Table 2). A significant amount of the variance of meaning in life was explained by the independent variables (F (4, 248) = 48.48, *p* < 0.001, *R*^2^ = 0.443, *R*^2^ Adjusted = 0.434). The analysis revealed that purpose β = 0.61, t (248) = 10.00, *p* > 0.001 and intrinsic aspirations β = 0.15, t (248) = 2.86, *p* = 0.005 both positively predicted meaning in life while extrinsic values negatively predicted meaning in life β = −0.12, t (248) = −2.30, *p* = 0.022. The relationship between self-efficacy and meaning in life was non-significant β = 0.004, t (248) = 0.06, *p* = 0.951.

To explore how meaning in life as well as its predictors shown from our initial regression analysis related to COVID-19 related stress, a further multiple regression was conducted with the variables entered simultaneously (a summary of these results is shown in Table 3). This revealed a significant model (F (5, 238) = 4.787, *p* = 0.001, *R*^2^ = 0.076, *R*^2^ Adjusted = 0.060); however, only meaning in life was significant in predicting less COVID-19 related stressors, β = −0.18, t (238) = −2.12, *p* = 0.035. Furthermore, against our expectations, intrinsic based goals did not significantly predict COVID-19 related stress β = 0.10, t (238) = 1.34, *p* = 0.167. The relationship between extrinsic values and COVID-19 related stressors was also non-significant β = 0.07, t (238) = 1.00, *p* = 0.317.

## 5. Discussion

Our findings suggested that a personal meaning in life might help buffer against the anxious and stress related reactions brought about by the COVID-19 pandemic. In line with expectations, a higher life meaning related negatively to the adverse cognitive reactions of people to the pandemic, suggesting a personal sense of meaning in life might help diminish stress responses when confronted with a challenging event. This replicated previous research showing that those with a high self-perceived meaning in life were more likely to cope well with distressing events [4,12,21]. Such research has extended to investigating the role life meaning plays in enduring personal traumas [12] as well as coping with collective adversity and stressful life events [21]. Specific to the COVID-19 pandemic, our findings augmented recent research showing meaning in life to be an effective coping mechanism for the psychological distress brought on by the pandemic [3,4,20]. Adding to this previous research, we also found that intrinsic based aspirations predicted higher meaning in life scores (versus extrinsic based aspirations, which did not). However, contrary to our hypothesis, these values were not significantly related to COVID-19 related stressors and therefore insignificant in mediating the relationship between a perceived meaning in life and the impact of COVID-19. This finding did still imply that intrinsic aspirations such as close relationships, community involvement and personal development may help in the construction of a meaningful life. This was in contrast to extrinsic based aspirations, which included the pursuit of wealth, fame and increased status that related negatively to life meaning. Whilst a body of research has indicated that intrinsic aspirations relate positively to wellbeing [26,34], further research is needed to explore how these factors may relate to meaning in life in times of stressful circumstances or events.

## 6. Limitations and Future Directions

A key limitation of our findings was they were based on correlational and cross-sectional data and therefore do not allow conclusions about the causal direction of the relationships. It is of course possible that people who were more threatened and stressed about the COVID-19 pandemic felt more bothered and apathetic by these experiences and consequently reported feeling less meaning in life. Obtaining direct experimental evidence showing that meaning in life may buffer against stressful events is needed to better understand these processes. A further limitation of our study was that the data collection was only completed at one time point during the COVID-19 pandemic and did not allow for a comparison prior to the pandemic, thus limiting the scope of the study. Future studies should endeavour to measure responses before and after the occurrence of a distressing event to allow for a comparison of responses. Lastly, our sample of participants was made up entirely of online participants, with our findings therefore limited by issues inherent with online data collection such as self-selection biases and generalizability.

Despite these limitations, our findings provided important further evidence to the understanding that life meaning can protect against the consequences of adverse events such as the COVID-19 pandemic. This supports past findings that have suggested that the presence of life meaning may help to put such societal threats into perspective, such that they become less motivationally salient while also providing a significant focus that draws ruminative attention away from threat related stimuli [14]. With research suggesting that the idea of purpose and meaning in life seems to be greater in developing contexts versus developed ones [16], more attention should be paid by clinicians and policy makers in developed countries towards the development of life meaning as an important protective factor against negative life events, as well as a key element of wellbeing and human flourishing. Furthermore, our findings hinted at the importance of intrinsic based goals including relationships, contributing to the community and personal growth as potentially important components to the development of a meaningful life.

## 7. Conclusions

The current study has added to the previous literature on coping resources employed during distressing events by showing the positive effect an individual’s perceived meaning in life has on protecting them from the impact of uncertain and negative life events. During times of personal as well as collective distress, a sense of life meaning can help people cope with trauma while also helping with the adjustment towards difficult circumstances such as a pandemic. A sense of life meaning centred on intrinsic goals may be particularly poignant in assisting with this. As Frankl [17] famously stated, man is ultimately searching for meaning and purpose in life above and beyond his immediate psychological, social and physical needs. When faced with particularly difficult times, pursuing life meaning seems to be a crucial yet understated factor in a person’s aptitude to cope.

## Figures and Tables

**Table 1:** Zero-order and partial correlations between measures.

	P	IA	EA	SEf	IC
**ML**	0.65 ***	0.27 ***	−0.07	0.44 ***	−0.24 ***
**P**		0.24 ***	0.05	0.64 ***	−0.21 **
**IA**			0.28 ***	0.34 ***	0.02
**EA**				0.14 *	0.12
**SEf**					−0.11

Note: ML = Meaning in Life, P = Purpose, IA = Intrinsic Aspirations, EA = Extrinsic Aspirations, SEf = Self-Efficacy, IC = Impact of COVID-19. *** *p* < 0.001, ** *p* < 0.01, * *p* < 0.05.

**Table 2:** Regression analyses of the relationships between meaning in life and the independent variables.

Variable	N	F	*R* ^2^	P	SEf	IA	EA
ML	248	48.48	0.43	0.61 ***	0.00	0.15 **	−0.12 *

Note: ML = Meaning in Life, P = Purpose, SEf = Self-Efficacy, IA = Intrinsic Aspirations, EA = Extrinsic Aspirations. *** *p* < 0.001, ** *p* < 0.01, * *p* < 0.05.

**Table 3:** Regression analyses of the relationships between meaning in life, its predictors and the impact of COVID-19 related stress.

Variable	N	F	*R* ^2^	ML	P	IA	EA
IC	238	4.78	0.06	−0.18 *	−0.11	0.09	0.07

Note: ML = Meaning in Life, IC = Impact of COVID-19, P = Purpose, IA= Intrinsic Aspirations, EA = Extrinsic Aspirations. * *p* < 0.05.

## Data Availability

The data that supports the findings of this study are available on request from the corresponding author.
